# Gene Expression and Protein Synthesis in Mitochondria Enhance the Duration of High-Speed Linear Motility in Boar Sperm

**DOI:** 10.3389/fphys.2019.00252

**Published:** 2019-03-12

**Authors:** Zhendong Zhu, Takashi Umehara, Tetsuji Okazaki, Masaaki Goto, Yoko Fujita, S. A. Masudul Hoque, Tomoko Kawai, Wenxian Zeng, Masayuki Shimada

**Affiliations:** ^1^Key Laboratory of Animal Genetics, Breeding and Reproduction of Shaanxi Province, College of Animal Science and Technology, Northwest A&F University, Yangling, China; ^2^Laboratory of Reproductive Endocrinology, Graduate School of Biosphere Science, Hiroshima University, Hiroshima, Japan; ^3^Livestock Research Institute, Oita Prefectural Agriculture, Forestry and Fisheries Research Center, Oita, Japan; ^4^Women’s Clinic Oizumi-Gakuenn, Tokyo, Japan; ^5^Department of Animal Breeding of Genetics, Bangabandhu Sheikh Mujibur Rahman Agricultural University, Gazipur, Bangladesh

**Keywords:** metabolic activity, glycolysis, mitochondrial oxidative phosphorylation, sperm motility pattern, energy source

## Abstract

Sperm motility patterns are continuously changed after ejaculation to fertilization in the female tract. Hyperactivated motility is induced with high glucose medium *in vitro* or the oviduct fluids *in vivo*, whereas sperm maintain linear motility in the seminal plasma or the uterine fluids containing low glucose. Therefore, it is estimated that sperm motility patterns are dependent on the energy sources, and the mitochondrial oxidative phosphorylation is activated to produce ATP in low glucose condition. To elucidate these hypotheses, boar sperm was incubated in different energy conditions with the transcription and translation inhibitors *in vitro*. Sperm motility parameters, mitochondrial activity, ATP level, gene expression and protein synthesis were analyzed. Sperm progressive motility and straight-line velocity were significantly increased with decreasing glucose level in the incubation medium. Moreover, the mitochondrial protein turnover meaning transcription and translation from mitochondrial genome in sperm is activated during incubation. Incubation of sperm with mitochondrial translation inhibitor (D-chloramphenicol) suppressed mitochondrial protein synthesis, mitochondrial activity and ATP level in sperm and consequently reduced the linear motility speed, but not the motility. Thus, it is revealed that the mitochondrial central dogma is active in sperm, and the high-speed linear motility is induced in low glucose condition via activating the mitochondrial activity for ATP generation.

## Introduction

Mammalian spermatozoa reside in the female genital tracts for several hours from ejaculation to fertilization process. Linear motility, as defined by low lateral amplitude and high straight-line velocity, is essential for sperm migration from the cervix to the uterus and then to oviduct (Shalgi et al., 1992; Suarez and Pacey, 2006). Just after ovulation, capacitated sperm leave the oviduct epithelial cells and move to the oocyte (Demott and Suarez, 1992; De Lamirande et al., 1997). The capacitated sperm show hyperactivated status (Suarez, 2008), defined as high curvilinear velocity as well as high lateral amplitude, and penetrate the oocyte for successful fertilization (Stauss et al., 1995). Sperm motility patterns are dependent on flagellar motion; the symmetry flagellar motion induces the linear motility pattern, whereas the asymmetry one is associated with hyperactivation (Phillips, 1972; Suarez et al., 1991; Ho et al., 2002; Curtis et al., 2012). The flagellum of mammalian sperm consists of nine fused pairs of microtubule doublets surrounding two central single microtubules (Fawcett, 1970; Woolley and Fawcett, 1973). Moreover, the sliding filament theory is adapted to sperm flagella, namely the sperm motility is induced by ATP production (Huxley and Niedergerke, 1954; Summers and Gibbons, 1971; Brokaw, 1972).

Glycolysis and mitochondrial oxidative phosphorylation (OXPHOS) are two main metabolic pathways for ATP production in mammalian cells. The metabolic pathways of cells are changed depending on the oxygen availability and the composition of metabolic substrates in their environment (Gohil et al., 2010; Potter et al., 2016). When HepG2 cells were cultured in the medium containing a high level of glucose, glycolysis pathway was highly activated; however, the reduction of glucose level in the medium switched off the glycolytic pathway to turn on OXPHOS pathway to generate ATP in the cells (Marroquin et al., 2007). Amino acids and pyruvate are the substrates of TCA cycle in mitochondria present at high concentrations in both seminal plasma and uterus fluids (Marden, 1961; Brown-Woodman and White, 1974; Harris et al., 2005). On the other hand, the high level of glucose is detected in the oviductal fluids, especially after ovulation (Nichol et al., 1992; Vecchio et al., 2007; Umehara et al., 2018). Therefore, for the successful fertilization *in vivo*, the sperm probably use the different metabolic pathways to produce ATP in each condition as similar to those in somatic cells (Ruiz-Pesini et al., 2007; Storey, 2008). It is generally accepted that the glycolysis pathway is essential for sperm hyperactivation (Du Plessis et al., 2015). However, there is limited information about the roles of mitochondria in producing ATP regarding sperm motility patterns.

Mitochondria, double membrane sub-cellular organelles have their own maternally inherited genome mitochondrial DNA (mtDNA) which encodes 13 polypeptides, 22 tRNAs, and 2 rRNAs. The polypeptides are essential subunits for mitochondrial electron transport chain (ETC) complexes wheras the tRNAs and rRNAs are essential for translation of the polypeptides (Anderson et al., 1981). The mitochondrial mRNA transcription (such as NADPH dehydrogenase subunits 1–6 (*mt-Nd1* – *mt-Nd6*), cytochrome c oxidase subunits 1–3 (*CoxI* – *CoxIII*), etc. and their translation processes are activated with increasing the mitochondrial activity and ATP level in serum-stimulated HeLa cells (Xiong et al., 2012). Therefore, we hypothesized that mitochondrial OXPHOS is activated in sperm for ATP supply, and their transcription and translation process become functioning to the sperm motility. Hence, the present study was performed to (1) determine whether sperm motility patterns and metabolic pathways in different glucose levels are changed or not, (2) understand the induction of gene expression and protein synthesis in sperm mitochondria, and (3) elucidate the roles of the mitochondrial transcription and translation in sperm motility.

## Materials and Methods

### Materials

Routine chemicals and reagents were obtained from Nakarai Chemical Co. (Osaka, Japan) or Sigma Chemical Co. (St. Louis, MO, United States).

### Animals and Semen Collection

Five healthy, fertile, and mature boars (between the ages of 2 and 4 years) were used in this study. The boars were housed individually, maintained under natural daylight, fed basal diets and been free access to water. The sperm-rich fraction was collected weekly from each boar using the gloved-hand technique. The sperm-rich fraction was filtered through double gauze.

### Ethics Statement

All animals and experimental procedures were treated in accordance with the National Institutes of Health Guide for the Care and Use of Laboratory Animals, approved by the Animal Care and Use Committee at Hiroshima University (approval number: E18-1).

### Sperm Incubation

Fresh sperm was diluted with Modena solution composed of 153 mM D-glucose, 26.7 mM trisodium citrate, 11.9 mM sodium hydrogen carbonate, 15.1 mM citric acid, 6.3 mM EDTA-2Na, 46.6 mM Tris, 1000 IU/mL penicillin G potassium and 1 mg/mL amikamycin (Okazaki et al., 2012). The concentration of glucose in Modena solution (153 mM) was defined as 100%. Lactose was used partially with glucose to make the different dose of glucose extender (153, 122.4, 91.8, 61.2, 30.6, and 0 mM; namely 100%, 80%, 60%, 40%, 20%, and 0%) because sperm did not use lactose as an energy substrate. Sperm were incubated for 1 h at 37°C in each media containing different levels of glucose. Some of the sperm were incubated with various concentrations of rotenone (0, 10, 100 nM, an inhibitor of complex I) for 3 h at 37°C in 30.6 mM glucose media (20% glucose). Other sperm were incubated with various concentrations of D-chloramphenicol (a mitochondrial translation inhibitor, CRP: 0, 200, 400, 600, 800 ng/mL), cycloheximide (mRNA translation inhibitor, CHX: 0, 50, 100 ng/mL) and α-amanitin (nuclear transcription inhibitor, AMNT: 0, 10, 50, 100 ng/mL) for 3 h at 37°C in 30.6 mM glucose media (20% glucose). Furthermore, sperm were also incubated in 30.6 mM glucose media (20% glucose) for up to 6 h at 37°C to evaluate whether the transcription and translation in sperm mitochondria is working or not.

### Evaluation of Sperm Motility by Computer-Assisted Sperm Analysis (CASA) System

After incubation of sperm in different treatments, a total of 10 μL of sample was placed in a pre-warmed counting chamber. According to our previous study (Umehara et al., 2018), sperm tracks (0.5 s, 45 frames) were captured at 60 Hz using a CASA system (HT CASA-Ceros II; Hamilton Thome, MA, United States). More than 200 individual trajectories were recorded. Non-progressive motility (%) = total motility (%) - progressive motility (%).

### Membrane Integrity

Membrane integrity was evaluated using a LIVE/DEAD Sperm Viability Kit (L7011; Thermo Fisher Scientific), as described previously (Zhu et al., 2017). Briefly, samples were stained with SYBR-14/PI. The staining was analyzed in a flow cytometer (FAC-S Calibur, BD Biosciences) with Excitation/Emission = 485/535 nm for SYBR-14 fluorescence, Excitation/Emission = 525/590 nm for PI fluorescence. A total of 50,000 sperm-specific events were analyzed. Data were processed by using the CellQuest program (BD Biosciences).

### Mitochondrial Activity

Sperm mitochondrial activity was measured with MitoPT^®^ JC-1 Assay Kit (911, ImmunoChemistry Technologies, llc.) according to Treulen et al. (2016). Briefly, sperm samples were incubated with 500 μL 1x working solution at 37°C for 30 min in dark, the mitochondrial activity was analyzed by flow cytometry using a filter with a bandwidth of 574/26 nm (Attune^®^NxT Acoustic Focusing Cytometer, Invitrogen) and measured as the mean fluorescence intensity (MFI) of JC-1 orange aggregates. A total of 50,000 sperm events were analyzed.

### Measurement of Sperm ATP Level

Enzylight^TM^ ATP Assay Kit (EATP-100, Bioassay System, Hayward, CA, United States) was used to detect sperm ATP level according to the manufacturer’s instruction. Briefly, sperm samples were mixed with assay buffer and substrates, and then the luminescence was measured with a luminometer (2030 Multilabel Reader ARVO X4; PerkinElmer Inc., Waltham, MA, United States). The ATP production was calculated over sperm concentration and expressed as nmol/10^7^ of sperm.

### RNA Extraction and Reverse Transcription-Polymerase Chain Reaction (RT-PCR)

Total RNA was obtained from fresh sperm and the granulosa cells (positive control) using the RNAeasy mini kit (QIAGEN Sciences, Germantown, MD, United States), according to the manufacturer’s instructions. Reverse transcription (RT) was performed using 500 ng poly-deoxythymidine and 0.25 U avian myeloblastosis virus reverse transcriptase (Promega, Madison, WI, United States) at 42°C for 75 min and 95°C for 5 min. RT-PCR analyses were performed with KOD FX Neo (TOYOBO Life Science, Osaka, Japan) according to the manufacturer’s instructions. Specific primers pairs used in the RT-PCRs are shown in Table 1; PCR products were resolved on 2% (wt/vol) agarose gels.

**Table 1:** List of primers employed for RT-PCR and the expected size.

Gene name	Primer sequences	Product size (Kb)
*mt-Nd1*	F: AATATGGCGAAAGGTCCGGC	104
	R: ACCCTAGCAGAAACCAACCG	
*mt-Nd2*	F: TGGCTAGGGCCATGGTTATT	152
	R: CCTAACACAAGCCACAGCCT	
*mt-Nd3*	F: GAGGCCTGCTGATCCTATCG	130
	R: AACCCTAGCCTCCCTACTCG	
*mt-Nd4*	F: AGGAGTGTTTGCAGTCCTCG	149
	R: TTGCCCACGGACTAACATCC	
*mt-Nd4L*	F: AGCTAGGGTGAAGTGTGTGT	127
	R: GATCGCCCTTGCAGGGTTAC	
*mt-Nd5*	F: GAAGGCGTAGGATACGGTGG	154
	R: CCCATTCGCCTCACTCACAT	
*mt-Nd6*	F: AAGCAGCAATCCCCATAGCTT	118
	R: GCGTTGAAGGAAGAGGAAGTAGA	
*Cytb*	F: TAGGGCCAACACTCCACCTA	115
	R: CACCCCAGCAAACCCACTAA	
*CoxI*	F: ACAGTTCATCCAGTACCCGC	169
	R: TCCCGATATGGCCTTTCCAC	
*CoxII*	F: GGCATGAAGCTGTGGTTTGA	108
	R: AGATGCTATCCCAGGACGACT	
*CoxIII*	F: ATACTCCTGAGGCGAGGAGG	132
	R: CCTAGCACCAACACCCGAAT	
*Atp6*	F: TTGGATCGAGATTGTGCGGT	188
	R: TGCCCCCACGATAATAGGAC	
*Atp8*	F: ATACCCAGCAAGCCCAGAAT	115
	R: GTGGGGGCAATAAAAGAGGCA	
*Ndufa7*	F: TTCCCAGCAGTCCTAGCGTA	126
	R: AGCTTCGCCTGTAAGTCTCG	
*Ndufb10*	F: ACCCTGTCACCTACCTTACGA	149
	R: GCACTCTGTGATGTCTGGCA	
*Ndufs4*	F: GGATCTTTGTTCCTGCTCGC	127
	R: GGATCAGCCGTTGATGACCA	


*mt-Nd1 – mt-Nd6, NADPH dehydrogenase subunits 1–6; *Cytb*, mitochondrially encoded cytochrome b; *CoxI* – *CoxIII*, cytochrome c oxidase subunits 1–3; *Atp6*, mitochondrially encoded ATP synthase 6; *Atp8*, mitochondrially encoded ATP synthase 8; *Ndufa7*, NADH-ubiquinone oxidoreductase subunit A7; *Ndufb10*, NADH-ubiquinone oxidoreductase subunit B10; *Ndufs4*, NADH-ubiquinone oxidoreductase subunit S4.*

### Quantitative PCR Analyses

Quantitative real-time PCR analyses were performed as previously (Shimada et al., 2008). Briefly, cDNA and primers shown in Table 1 were added to 15 μL total reaction volume of the Power SYBR Green PCR master mix (Applied Biosystems). PCRs were then performed using the StepOne real-time PCR system (Applied Biosystems).

### Immunofluorescence

Sperm cells were washed with PBS and fixed with 4% paraformaldehyde for 5 min. The sperm were spread onto glass slides, then permeabilized by 0.5% Triton X-100 in PBS. Non-specific binding was blocked with PBS contained 10% bovine serum albumin (w/v) (Life Technologies, Grand Island, NY, United States) for 30 min at room temperature. Sperm were proved with a 1:100-diluted NADPH dehydrogenase subunits 1 (MT-ND1) antibody (1973-1-AP, Proteintech, United States), NADPH dehydrogenase subunits 6 (MT-ND6) antibody (bs-3955R; Bioss, Inc., Boston, MA, United States), and NADH-ubiquinone oxidoreductase subunit A7 (NDUFA7) antibody (ab140871; Abcam, United States). The antigens were visualized with biotinylated goat anti-rabbit Cy3-IgG or donkey anti-goat FITC-IgG (1:200). Subsequently, sperm were incubated with 4^′^,6^′^-diamidino-2-phenylindole (DAPI; 5 μg/mL; Sigma-Aldrich). Digital images were captured using a BZ-9000 microscope (Keyence Co., Osaka, Japan). Negative control was prepared at the same time without the primary antibody.

### Western Blotting

Western blotting analyses were performed according to our previous study (Umehara et al., 2018). Total protein was extracted from sperm in sodium dodecyl sulfate (SDS) sample buffer. The protein was separated by 12.5% SDS-PAGE and transferred to PVDF blotting membrane (GE Bioscience, Newark, NJ, United States). Non-specific binding sites were blocked by incubation in Tris-buffered saline (TBS) containing 0.1% (v/v) Tween-20 and 5% (w/v) bovine serum albumin (Life Technologies, Grand Island, NY, United States). The membranes were immunoblotted with primary antibodies [anti-MT-ND1, anti-MT-ND6, anti-NDUFA7 and anti-α-tubulin (2148; Cell Signaling Technology, Inc.)] diluted in 5% bovine serum albumin in TBS-Tween (1:1000 dilution) overnight at 4°C. Followed by incubation with HRP conjugated secondary [goat anti-rabbit antibody (7074S, Cell Signaling Technology, Inc.) for MT-ND1, MT-ND6, and α-tubulin, donkey anti-goat (ab97110, Abcam, United States) for NDUFA7, 1:5000 dilution]. After washing in TBST, enhanced chemiluminescence (ECL) detection was performed by using the ECL^TM^ Prime Western Blotting Detection Reagents (RPN2235, GE Bioscience) according to the manufacturer’s specifications, and appropriate exposure of blots to Fuji X-ray film (Fujifilm, Tokyo, Japan). Band intensities were analyzed using a Gel-Pro Analyzer (Media Cybernetics, Rockville, MD, United States).

### Statistical Analysis

All data were tested for normality and variance homogeneity prior to statistical analysis. Data were transformed by arc-sin square root transformation when it is necessary. Data from three replicates for comparison were performed by either Student’s *t*-test or one-way analysis of variance followed by Tukey’s *post hoc* test (Statview; Abacus Concepts, Inc., Berkeley, CA, United States). All the values are presented as the mean ± standard error (SE). Treatments were considered statistically different from one another at *p* < 0.05.

## Results

### Low Glucose Condition Increases the Mitochondrial Activity and Motility Patterns of Sperm at 1 h Incubation

**FIGURE 1:**
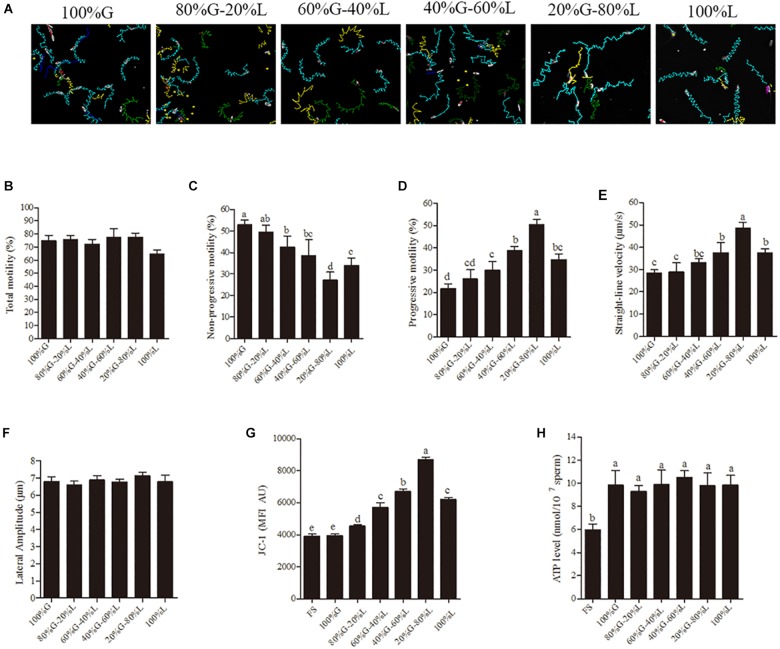
Low glucose condition improves the sperm motility patterns through enhancing the mitochondrial activity at 1 h of incubation. **(A)** CASA derived changes in the sperm motility track from circular to linear by reducing the glucose level from 153 mM to 0 mM (namely 100–0%). **(B–F)** Dynamic changes in the sperm parameters: **(B)** total motility, **(C)** non-progressive motility **(D)** progressive motility, **(E)** straight-line velocity and **(F)** lateral amplitude. **(G)** Kinetic changes in the mitochondrial activity. **(H)** ATP level in the sperm. Values are specified as mean ± standard error of mean (SEM) of three replicates. Columns with different lowercase letters differ significantly (*p* < 0.05). The letters in *X*-axis are symbolized as – G, glucose; L, lactose; FS, fresh sperm.

The sperm motility tracks generated by CASA revealed that the incubation in high glucose media made circle-like tracks, while low glucose condition made it linear-like tracks (Figure 1A). The sperm total motility wasn’t significantly changed in all glucose concentration [from 30.6 mM (20%) to 153 mM (100%)] in the media (Figure 1B). However, the reduction of glucose level from 153 mM to 30.6 mM significantly increased the sperm progressive motility and straight-line velocity, decreased the non-progressive motility in a dose-dependent manner (Figure 1A,C–E). The high linier motility was observed until 3 h; however, the total motility, progressive motility, and straight-line velocity were significantly decreased at 6 h of incubation (Supplementary Figure 2). Meanwhile, no significant difference was observed in lateral amplitude (Figure 1F), an index for evaluating hyperactivation among the treatment groups. The mitochondrial activity was also significantly increased by the reduction of glucose level in the incubation medium (Figure 1G and Supplementary Figure 1). However, the ATP level showed no significant difference among the treatment groups (Figure 1H).

### Addition of Rotenone to Incubation Media Reduces the Mitochondria Activity, ATP Level and Kinetic Patterns of Sperm

**FIGURE 2:**
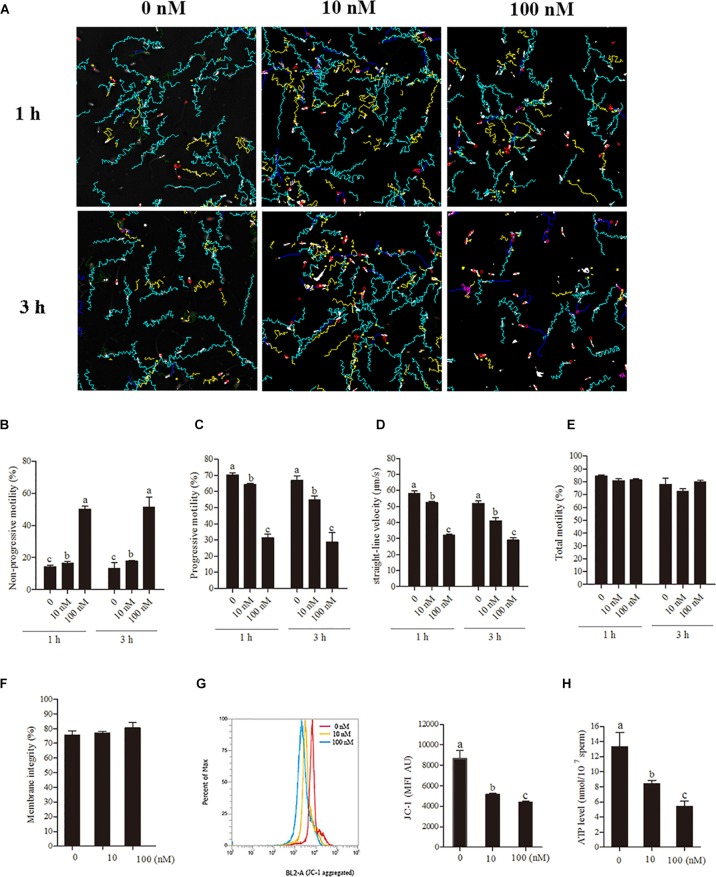
Sperm incubated with rotenone in 30.6 mM glucose media alters the mitochondrial activity, ATP level and kinetic patterns. **(A–E)** Kinetic changes in the sperm motility patterns at 1 and 3 h of incubation, **(A)** motility tracks generated from CASA, **(B)** non-progressive motility, **(C)** progressive motility, **(D)** straight-line velocity, **(E)** total motility. **(F–H)** Rotenone-induced changes in mitochondrial attributes at 1 h of incubation, **(F)** membrane integrity, **(G)** mitochondrial activity, and **(H)** ATP levels. Values are specified as mean ± SEM of three replicates. Columns with different lowercase letters differ significantly (*p* < 0.05).

To understand the relationship between sperm motility pattern and mitochondrial ATP production, rotenone (an inhibitor of complex I) was added to the low glucose medium [30.6 mM (20%) glucose]. The sperm motility tracks and the CASA analytic data revealed that the progressive motility and straight-line velocity were significantly decreased with the treatment with rotenone at 1- and 3-h points in a dose-dependent manner, whereas the non-progressive motility was increased (Figure 2A–D). However, the addition of rotenone did not alter sperm total motility during incubation (Figure 2E). Apart from the motility data, the sperm membrane integrity used for survival evaluation showed no significant difference among the treatment groups (Figure 2F). The mitochondrial activity and ATP levels were significantly reduced by the addition of rotenone in a dose-dependent manner (Figure 2G,H) at 1-h point.

### Transcription and Translation in Sperm Mitochondria Is Working During Incubation

**FIGURE 3:**
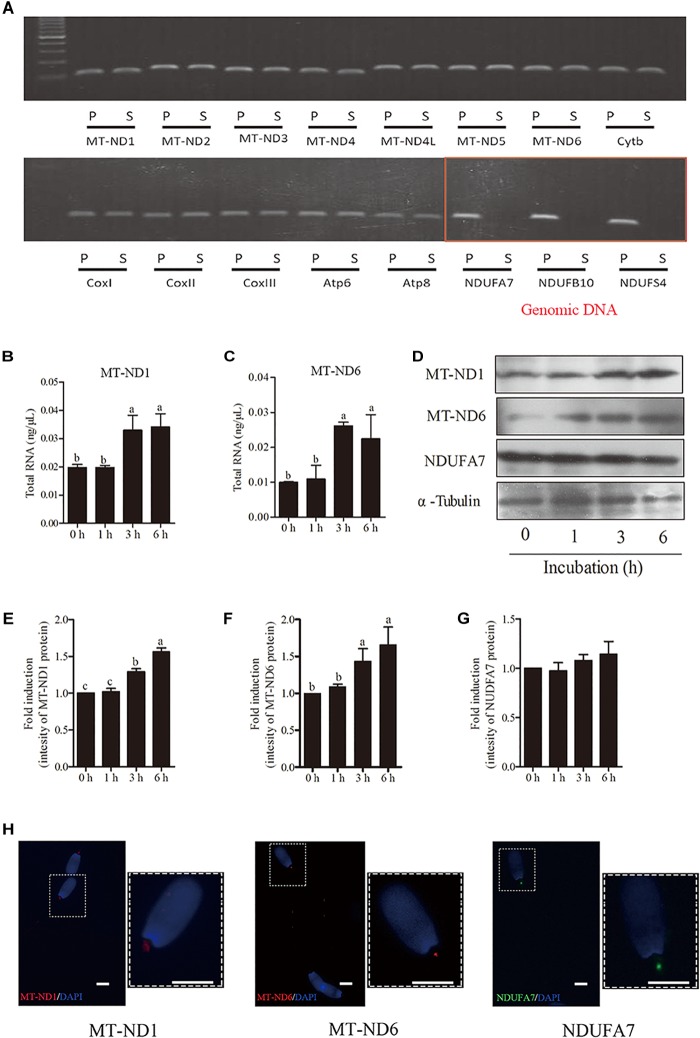
The transcription and translation in sperm mitochondria. **(A)** RT-PCR derived expression of mitochondrial and nuclear genes, where – S, boar sperm; P, positive control (porcine ovarian granulosa cell). **(B,C)** Time-dependent changes in the expression of *mt-Nd1* **(B)** and *mt-Nd6* **(C)** genes during 6 h incubation. **(D)** Western blotting image showing the expression of the mitochondria-encoded protein (MT-ND1 and MT-ND6), nuclear-encoded protein (NDUFA7), and α-tubulin during 6 h incubation. **(E–G)** Quantitative expression of the MT-ND1, MT-ND6, and NDUFA7 over α-tubulin (control) generated from western blotting. **(H)** Immunolocalizations of MT-ND1, MT-ND6, and NDUFA7 in boar sperm, scale bar indicates 5 μm. Values are specified as mean ± SEM of three replicates. Columns with different lowercase letters differ significantly (*p* < 0.05).

To elucidate whether the transcription and translation in sperm mitochondria is working or not, we checked the expression of 13 genes derived from mtDNA and 3 genes derived from nuclear DNA in sperm. Granulosa cells were used as the positive control. We detected all of the 13 mitochondrial-encoded mRNAs in both sperm and granulosa cells. However, the nucleus-encoded mRNAs of NDUFA7, NDUFB10, and NDUFS4 were not detected in sperm by 40-cycles PCR, although strongly expressed in granulosa cell (Figure 3A). Moreover, the expression of genes *mt-Nd1* and *mt-Nd6* were significantly increased at 3-h and 6-h points, but no change at 1-h point (Figure 3B,C). Similarly, western blot positive bands of MT-ND1 and MT-ND6 showed that the intensities of mitochondrial proteins were significantly increased at 3 h and 6 h but not changed at 1-h point (Figure 3D–F, *p* < 0.05). Meanwhile, the signal of the nuclear encoded NDUFA7 protein was not changed throughout the 6-h incubation period (Figure 3D,G). We also observed that the immunolocalizations of MT-ND1, MT-ND6, and NDUFA7 were in the sperm midpiece which also the location of mitochondria (Figure 3H).

### Mitochondrial Translational Inhibitor (CRP) Reduces the Mitochondrial Activity, ATP Level, Protein Synthesis, as Well as Motility Patterns of Sperm

**FIGURE 4:**
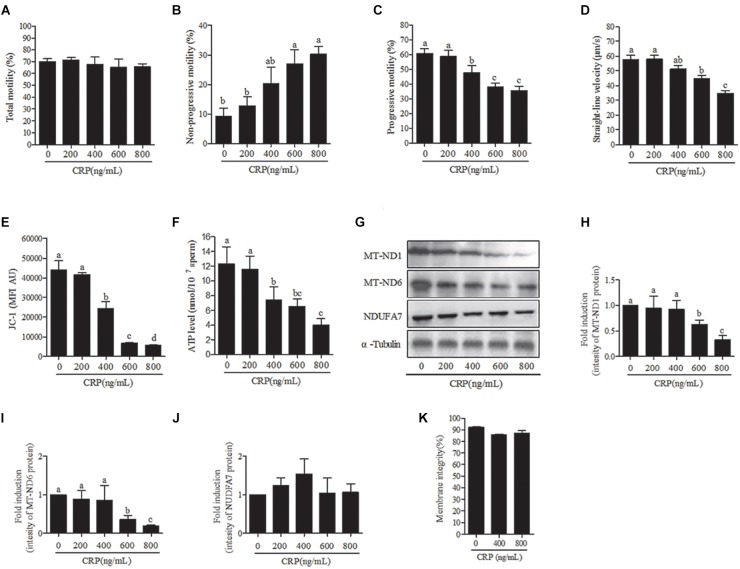
Effect of D-chloramphenicol (CRP) on boar sperm at 3 h incubation with different doses (0, 200, 400, 600, 800 ng/mL). **(A–F)** Dose-dependent changes in the sperm **(A)** total motility, **(B)** non-progressive motility, **(C)** progressive motility, **(D)** straight-line velocity, **(E)** mitochondrial activity, and **(F)** ATP level. **(G)** Western blotting image showing the expression of the MT-ND1, MT-ND6, NDUFA7, and α-tubulin. **(H–J)** Quantitative expression of the MT-ND1, MT-ND6, and NDUFA7 over α-tubulin (control) generated from western blotting. **(K)** Membrane integrity measured in sperm. Values are means ± SEM of three replicates. Columns with different lowercase letters differ significantly (*p* < 0.05).

Sperm were incubated with various doses (0, 200, 400, 600, 800 ng/mL) of CRP, a specific mitochondrial translational inhibitor for 3 h. At 3 h point, the progressive motility, straight-line velocity, mitochondrial activity and ATP levels were significantly decreased at 400, 600, and 800 ng/mL doses (Figure 4A,C–F) while the non-progressive motility was increased (Figure 4B). Moreover, the intensity of MT-ND1 and MT-ND6 proteins are observed to be significantly decreased only at higher (600 and 800 ng/mL) doses (Figure 4G–J). The sperm membrane integrity showed no significant difference (Figure 4K).

**FIGURE 5:**
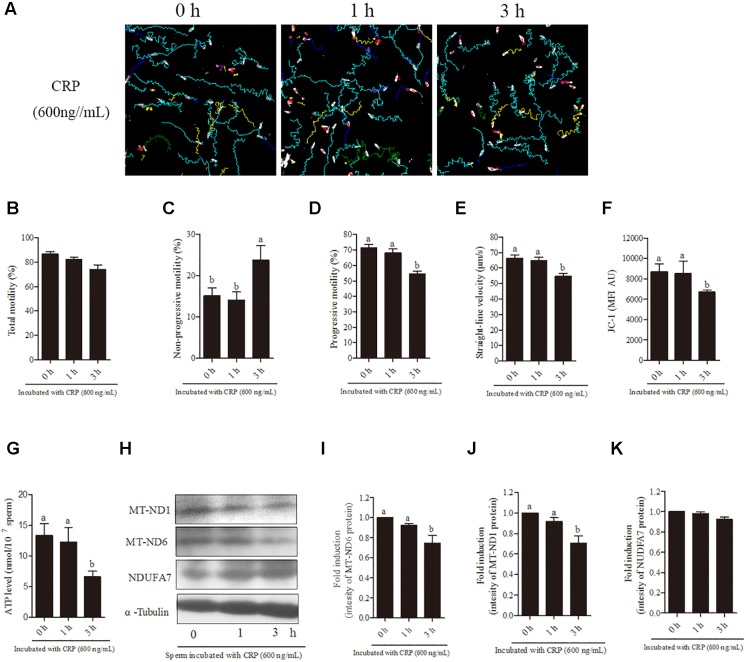
Effect of 600 ng/mL CRP on boar sperm during 3 h incubation in 30.6 mM glucose media. **(A)** Changes in the motility tracks of sperm generated by CASA system. **(B–G)** Time-dependent changes in the sperm **(B)** total motility, **(C)** non-progressive motility **(D)** progressive motility, **(E)** straight-line velocity, **(F)** mitochondrial activity, and **(G)** ATP level. **(H)** Western blotting image showing the expression of the MT-ND1, MT-ND6, NDUFA7, and α-tubulin. **(I–K)** Quantitative expression of the MT-ND1, MT-ND6, and NDUFA7 over α-tubulin (control) generated from western blotting. Values are means ± SEM of three replicates. Columns with different lowercase letters differ significantly (*p* < 0.05).

In terms of time-dependent experiment, a significant (*p* < 0.05) decrease was observed with 600 ng/mL CRP in the sperm progressive motility, straight-line velocity (Figure 5A,D,E), mitochondrial activity and ATP levels (Figure 5F,G) at 3 h of incubation point but not at 1-h point. Moreover, addition of 600 ng/mL CRP significantly increased non-progressive motility at 3-h point incubation (Figure 5C). However, the total motility was not changed with addition of 600 ng/mL CRP both at 1-h and 3-h points (Figure 5B). Furthermore, when sperm were incubated with 600 ng/mL CRP, the intensity of MT-ND1 and MT-ND6 proteins was significantly decreased at 3-h point but not changed at 1-h point (Figure 5H–J, *p* < 0.05), whereas the NDUFA7 protein signal was not changed during incubation (Figure 5H,K).

### Cytoplasmic Translation Inhibitor (CHX) and Nuclear Transcription Inhibitor (AMNT) Do Not Affect the Sperm Quality

**FIGURE 6:**
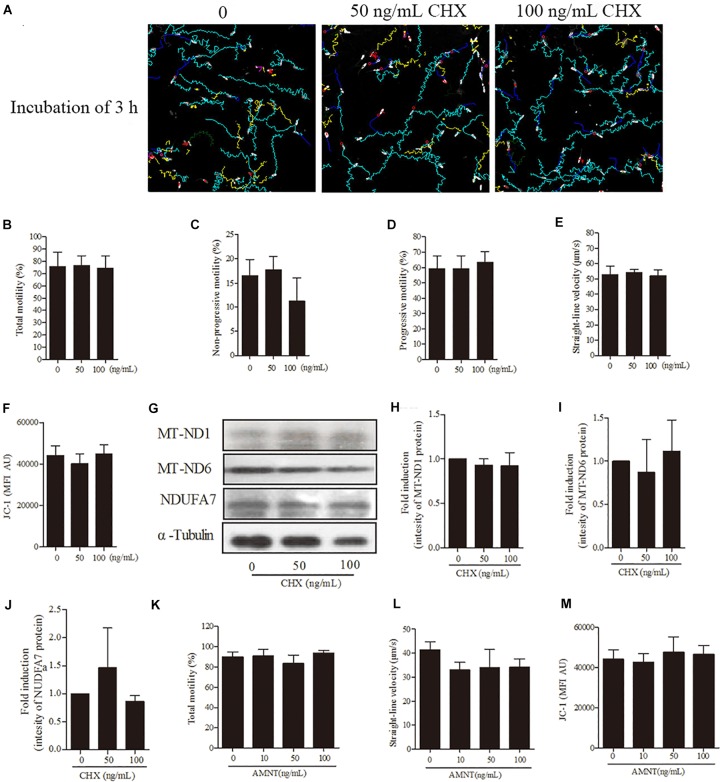
Effects of cycloheximide (CHX) and α-amanitin (AMNT) on boar sperm at 3 h of incubation 30.6 mM glucose media. **(A)** Tracks of sperm motility incubated with different concentrations of CHX (0, 50, and 100 ng/mL) generated by CASA system. **(B–E)** Motility patterns: **(B)** total motility, **(C)** non-progressive motility, **(D)** progressive motility, **(E)** straight-line velocity. **(F)** Mitochondrial activity. **(G)** Western blotting image showing the expression of the MT-ND1, MT-ND6, NDUFA7, and α-tubulin. **(H–J)** Quantitative expression of the MT-ND1, MT-ND6, and NDUFA7 over α-tubulin (control) generated from western blotting. **(K)** Total motility, **(L)** straight-line velocity, and **(M)** mitochondrial activity of sperm were not changed after 3 h of incubation with different concentrations of AMNT (0, 10, 50, and 100 ng/mL). Values are means ± SE of three replicates. Columns with different lowercase letters differ significantly (*p* < 0.05).

For further confirmation of our hypothesis that sperm linear motility patterns are solely dependent on transcription and translation in mitochondria but not in nucleus and cytoplasm, we incubated sperm with CHX and AMNT for 3 h. However, sperm incubated with different doses (0, 50, 100 ng/mL) of CHX revealed that the sperm motility tracks were unchanged (Figure 6A). Moreover, no significant difference was observed among the treatment doses in motility patterns [total motility, non-progressive motility, progressive motility, and straight-line velocity (Figure 6B–E)], mitochondrial activity (Figure 6F) and the intensity of the proteins (MT-ND1, MT-ND6, and NDUFA7) (Figure 6G–J). Similarly, sperm incubated with AMNT showed a non-significant difference in the total motility, straight-line velocity and mitochondrial activity (Figure 6K–M).

## Discussion

*In vivo* fertilization, sperm are ejaculated to the female reproductive tract with seminal plasma and swim toward oviduct for the fertilization (Suarez and Pacey, 2006). During the journey of sperm in female reproductive tracts, the motility pattern of sperm is altered from linear motility in the uterus to zigzag motility in the oviduct (De Lamirande et al., 1997). It is well known that the sperm motility is regulated by ATP produced either from glycolytic pathway in cytoplasm or OXPHOS in mitochondria or both (Storey, 2008; Mukai and Travis, 2012; Du Plessis et al., 2015). Especially, Odet et al. (2008) demonstrated that the glycolysis was important for hyperactivated motility using *Ldh-c* knock out mice model. On the other hand, *in vitro* incubation in low-glucose or glucose-free BWW medium with rotenone, an inhibitor of complex I, decreased the sperm progressive motility and straight-line velocity in stallion (Plaza Davila et al., 2015) and human (Barbonetti et al., 2010), suggesting that mitochondrial ATP production is also important for sperm motility. However, the different roles of metabolomics pathways in sperm motility remain unclear. In this study, using the medium containing different doses of glucose we revealed that mitochondrial activity was increased under low glucose condition, and the ATP produced in mitochondria was associated with a high-speed linear motility in boar sperm.

Mitochondria have several functions, such as cell calcium homoeostasis (Contreras et al., 2010), lipid homoeostasis (Labbe et al., 2014), release of cytochrome c (Wang, 2001) and the ATP synthesis via OXPHOS (Bratic and Trifunovic, 2010), etc. Especially, the production of ATP in mitochondria is essential for cell survival and cell homeostasis (Osellame et al., 2012). However, during the process of ATP production in mitochondria, reactive oxygen species (ROS) are produced as the by-products, and then the excessive accumulation of ROS reduces the production of mitochondrial ATP via enzyme degradation. The mitochondrial (TC) complexes, including NADH dehydrogenase (complex I), succinate dehydrogenase/fumarate reductase (complex II), cytochrome c reductase (complex III), cytochrome c oxidase (complex IV) and ATP synthase (complex V) are required for ATP production in mitochondria (Lenaz and Genova, 2010). In addition, the 13 mtDNA-encoded polypeptides are partly subunits of complex I, III, IV, and V (Chomyn et al., 1985; Amaral et al., 2013). Thus, to keep ATP level in sperm, the gene expression and protein synthesis in mitochondria would be required.

To elucidate the function of mitochondrial transcription and translation in sperm, the expression and protein synthesis in mitochondria were examined in our study. The genes encoding mitochondria were highly expressed in sperm but the genes encoding nuclear genome were not detected during incubation. The protein turnover system in mitochondria was essential for sperm motility when the sperm were incubated in low glucose media. Gur and Breitbart (2006) showed that the addition of D-chloramphenicol, a mitochondrial translation inhibitor, in modified Tyrode’s glucose-free medium suppressed protein synthesis in bull sperm. Moreover, transcripts of all the mitochondrial genes were detected in mouse epididymal sperm (Alcivar et al., 1989). Interestingly, the cycloheximide (CHX), an inhibitor to cytoplasmic translation did not suppress the intensity of proteins encoded by not only mtDNA, but also by nuclear genome in this study, indicating that the nuclear genome encoded protein is more stable and resistant to oxidative stress during incubation. In addition, when HeLa cells were cultured with a ROS generator, the levels of mitochondrial encoded proteins (MT-ND6, MT-ND1, and MT-CYB) were significantly decreased, but the HSP9A levels, a nuclear-encoded and mitochondria-localized protein showed no significant change (Xie et al., 2012). About 92% of proteins in mammals have at least one cysteine residue containing exposed thiol which is free to interact with the aqueous solvent (Miseta and Csutora, 2000). Requejo et al. (2010) reported that mammalian mitochondria had a large number of redox-active exposed thiols on the surface of native proteins, especially the 75-kDa subunit (NDUFS1) complex I that encoded by nucleus (Hurd et al., 2008). Moreover, the cysteine residue of mitochondrial encoded protein is comparatively lower than those of nuclear-encoded protein in the mitochondrial ETC complexes (cysteine residue per protein:1.69 vs. 3.56; cysteine residue% of protein sequence: 0.773 vs. 1.667%; the data were counted from the protein sequence in GeneCards Database^1^), signifying that the resistant performance for scavenging ROS of mitochondrial-encoded protein is lower than that in nuclear-encoded protein. Additionally, post-translational modifications including the formation of sulfenic acids (Charles et al., 2007) and *S*-nitrosothiols (Hogg, 2002) or glutathionylation (Cotgreave and Gerdes, 1998) to the protein thiols are important to maintain cellular redox signaling and protein stability. Usually, most of the nuclear-encoded proteins in mitochondria are potentially post-translationally modified in the cytoplasm prior to translocate to the mitochondria (Hofer and Wenz, 2014), suggesting that the mitochondrial-encoded proteins were more sensitive to ROS and unstable than nuclear-encoded proteins in sperm. Therefore, to keep the ATP production and the linear motility pattern, the transcription and translation in mitochondria are activated during incubation and may also be in the uterus for the successful *in vivo* fertilization.

In this study, the mitochondria activity and high-speed linear motility similar to uterus were up-regulated with the reduction of glucose concentration. Previous study showed that the concentration of glucose in the mice uterus is lower than those in oviduct during fertilization process (Harris et al., 2005). Additionally, sperm linear motility is induced in the semen plasma and uterus (De Lamirande et al., 1997), suggesting that sperm mitochondria would be activated in uterus when it was presented in the low glucose uterus fluid. However, when sperm was injected to uterus in artificial insemination technique, the sperm was diluted with human tubal fluid (HTF) medium that is commonly used for *in vitro* fertilization in human and with Modena solution that contains a high dose of glucose in pig. The artificial insemination technique is commercially applied worldwide to breed pigs; however, the technique is still not efficient, as the large sperm numbers per sow in estrus is required for getting high reproductive performance as similar to that by natural mating (5–7 × 10^9^ sperm for per sow fertilization) (Knox, 2016). The large sperm numbers are a critical limitation for artificial insemination application not only in pigs but also in human infertility treatment. Because in human artificial insemination, 1 × 10^7^ or more motile sperm are required (Van Voorhis et al., 2001), *in vitro* fertilization or intracytoplasmic sperm injection (ICSI) are usually selected in the case of oligospermia. Our novel insight in which high-speed linear motility is induced by low glucose medium might provide a new strategy for improving the artificial insemination technique of both livestock animals and human infertility care.

## Conclusion

In conclusion, the transcription and translation in sperm mitochondria is active and the low glucose condition improves sperm progressive motility, straight-line velocity and mitochondrial activity during incubation *in vitro*. Reduction of the glucose level in diluted medium enhances the duration of high-speed linear motility via activating the mitochondrial activity for ATP generation in the present study. Thus, high-speed linear motility induced by low glucose medium is a novel factor that improves the fertilization of artificial insemination in livestock animals and human infertility care. It is possible that diluting sperm with low glucose insemination medium will enhance sperm high-speed linear motility in the female genital tracts to improve fertilization as a simple and low-cost approach.

## Data Availability

All datasets generated for this study are included in the manuscript and/or the Supplementary Files.

## Author Contributions

ZZ and TU were responsible for experimental design, sample, data analysis, and writing the manuscript. TO, MG, YF, and TK collected the samples and interpreted the data. SH wrote the manuscript and edited it. WZ were responsible for discussion about experimental design, data analysis, and writing the manuscript. MS supervised all aspects of this study and wrote the manuscript.

## Conflict of Interest Statement

The authors declare that the research was conducted in the absence of any commercial or financial relationships that could be construed as a potential conflict of interest.

## Footnotes

1 https://www.genecards.org
