# Supplementary material for: Gene Expression and Protein Synthesis in Mitochondria Enhance the Duration of High-Speed Linear Motility in Boar Sperm
Source: Front Physiol. 2019 Mar 12;10:252. doi: 10.3389/fphys.2019.00252 (PMC6422996; doi:10.3389/fphys.2019.00252)
Supplement: Supplementary file 1 [file Table_1.DOCX]

**Supplementary Information**


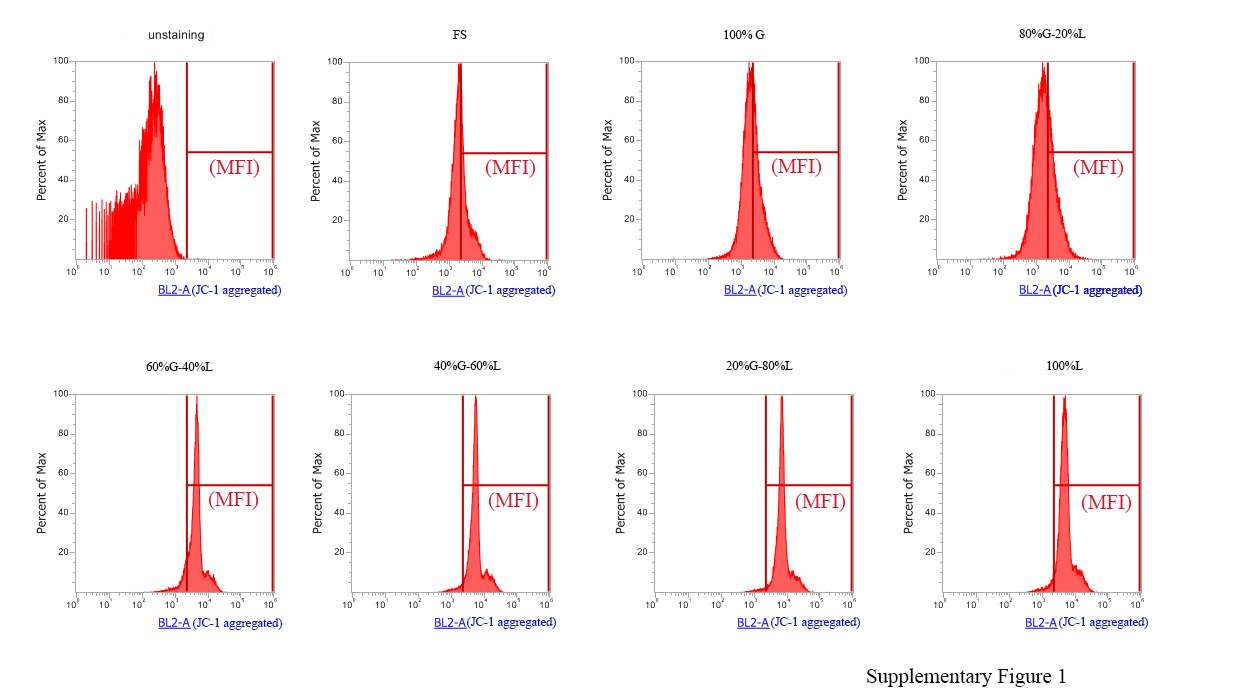


**Supplementary Figure 1.** Reduced glucose level increased sperm mitochondrial activity. Sperm mitochondrial activity was measured with the MitoPT® JC-1 Assay Kit, the fluorescence of JC-1 orange aggregates was analyzed by flow cytometry using a filter with a bandwidth of 574/26 nm. Mean fluorescence intensity (MFI) of JC-1 orange aggregates were used to analyzed.


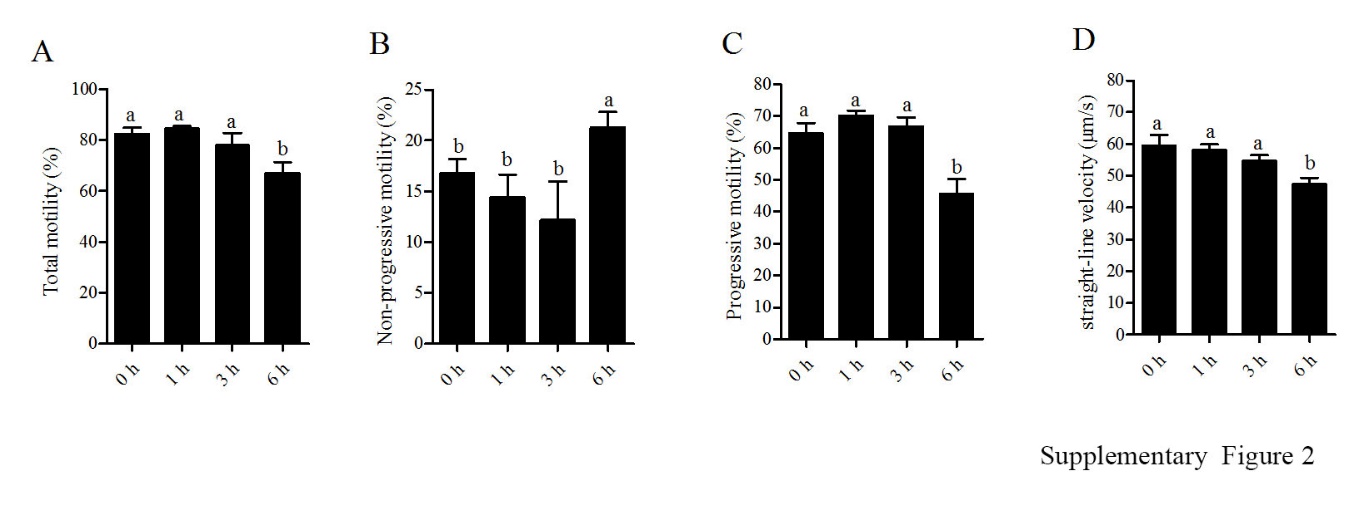


**Supplementary Figure 2.** Kinetic changes in the sperm motility patterns during 6 h of incubation in 30.6 mM glucose media. (A) total motility, (B) non-progressive motility, (C) progressive motility, (D) straight-line velocity.
